# *Brucella* osteoarthritis: recent progress and future directions

**DOI:** 10.3389/fmicb.2025.1522537

**Published:** 2025-02-04

**Authors:** Jinlei Chen, Feijie Zhi, Guanghai Zhao, Mengru Su, Hao Geng, Wei Song, Yuefeng Chu, Haihong Zhang

**Affiliations:** ^1^Department of Orthopedics, Lanzhou University Second Hospital, Lanzhou, China; ^2^Orthopaedics Key Laboratory of Gansu Province, Lanzhou, China; ^3^State Key Laboratory for Animal Disease Control and Prevention, College of Veterinary Medicine, Lanzhou Veterinary Research Institute, Chinese Academy of Agricultural Sciences, Lanzhou University, Lanzhou, China; ^4^Gansu Province Research Center for Basic Disciplines of Pathogen Biology, Lanzhou, China; ^5^Key Laboratory of Veterinary Etiological Biology, Key Laboratory of Ruminant Disease Prevention and Control (West), Ministry of Agricultural and Rural Affairs, Lanzhou, China

**Keywords:** osteoarthritis, *Brucella*, animal models, pathophysiological mechanisms, inflammation

## Abstract

Brucellosis is a common zoonosis, and *Brucella* osteoarthritis is the most common chronic complication of brucellosis. Development of brucellosis osteoarthritis involves multiple organs, tissues, and cells. *Brucella* grows and multiplies in intrinsic cells of the skeleton, including osteoblasts, osteocyte and osteoclasts, which results in sustained release of bacteria that leads to exacerbation of the immune response. Concurrently, activation of the immune system caused by invasion with *Brucella* may affect the dynamic balance of the skeleton. A variety of *in vitro* and *in vivo* models have been employed to study *Brucella* osteoarthritis, such as using bone marrow-derived macrophages to establish cell models and mice to develop animal models of *Brucella* osteoarthritis. However, limited studies on the molecular pathological mechanisms of *Brucella* osteoarthritis have been performed and inadequate animal models have been developed due to the challenging parameters of *Brucella* research. This paper reviews recent advances in the clinical features, molecular pathological mechanisms, and animal models of *Brucella* osteoarticular infections. This review underscores the complexity of the pathogenesis of *Brucella* osteoarticular infections and highlights inflammation as a contributing factor to bone loss caused by *Brucella*. Additionally, the significant proliferation of *Brucella* in skeletal resident cells also is an important factor leading to bone loss. A deeper understanding of the molecular pathological mechanism of *Brucella* osteoarthrosis and their animal models could provide robust support for the prevention and treatment of *Brucella* osteoarticular disease.

## Introduction

*Brucella* is a facultative, intracellular Gram-negative coccus that infects humans, wild animals, and livestock (Yagupsky et al., 2020). Six classical species of *Brucella* have been identified in the past 40 years, among which the most common in livestock and humans are *Brucella melitensis* (primary host are sheep and goats), *Brucella abortus* (major host is cows), and *Brucella suis* (infects swine, reindeer, and hares) (About et al., 2023). Human brucellosis is a zoonotic disease caused by the bacterium. The global annual incidence of human brucellosis is 2.1 million according to evidence-based conservative estimates, with most risks and cases concentrated in Africa and Asia (Laine et al., 2023). Infection causes subacute or chronic debilitating diseases with non-specific clinical manifestations and usually is associated with (i) contact with *Brucella*-infected livestock or wild animals, (ii) intake of unpasteurized dairy products, (iii) handling of infected livestock or laboratory animals (occupational hazards), or, (iv) exposure to live attenuated vaccines (Clapp et al., 2011; Noviello et al., 2004). Although human brucellosis rarely is fatal, the condition may severely debilitate and cripple. Acute brucellosis is associated with nonspecific influenza-like symptoms, including intermittent fever, headache, discomfort, back pain, and myalgia (Wang et al., 2022). The pathological manifestations of chronic brucellosis vary and encompass arthritis, spondylitis, endocarditis, meningitis, and long-term fatigue. Brucellosis is treated mainly with diverse antibiotics despite which recurrence still occurs over time (Qureshi et al., 2023).

*Brucella* osteoarthritis affects up to 85% of patients and is the most common clinical manifestation of human brucellosis (Scian et al., 2013). The most prevalent forms of *Brucella* osteoarthritis are sacroiliac arthritis, spondylitis, and peripheral arthritis (Giambartolomei et al., 2017) which can be caused by different *Brucella* species (González-Gay and García-Porrúa, 2014). Bone and joint involvement may be observed in patients with chronic brucellosis and may occur at any age. *Brucella* osteoarthritis causes cartilage loss and bone erosion in different joints (Khalaf et al., 2020; Wang et al., 2022).

**Figure 1:**
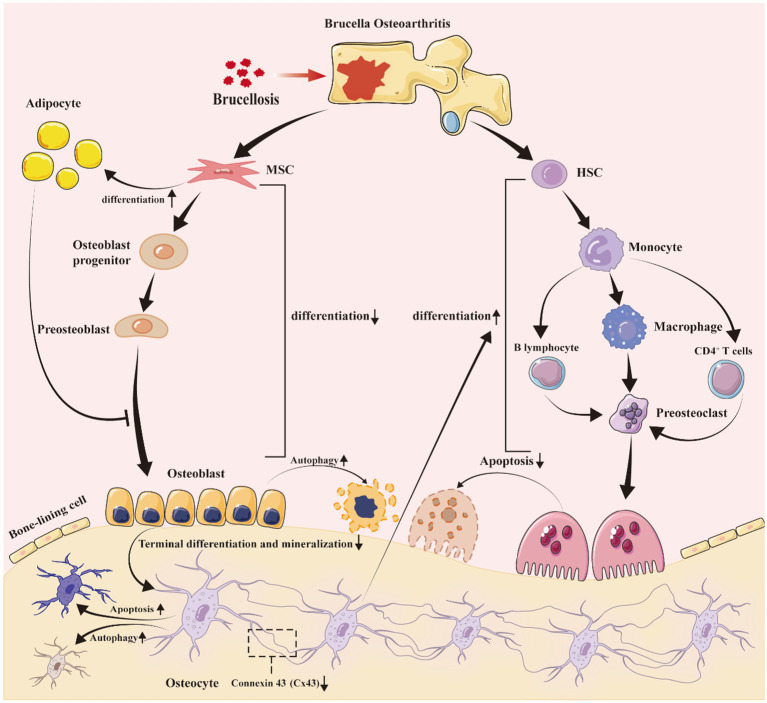
*Brucella* infection disrupts bone homeostasis, resulting in bone defects. After *Brucella* infection, the bacteria colonize in bone joints, creating a long-term chronic inflammatory environment. This environment, along with the direct and indirect effects of *Brucella* on osteogenic progenitor cells and infiltrating cells, promotes: (1) a reduction in the differentiation of mesenchymal stem cells into osteoblasts, while increasing their differentiation into adipocytes; (2) the increased adipocytes secrete inflammatory factors following *Brucella* infection, further exacerbating the inhibition of osteoblast differentiation; (3) a decrease in the terminal differentiation and mineralization of osteoblasts, impacting the formation of bone cells; (4) alterations in the expression of gap junction protein 43 in bone cells, leading to increased apoptosis and autophagy; (5) an elevated secretion of inflammatory mediators by bone cells, enhancing the differentiation and maturation of osteoclasts; (6) an increase in pro-inflammatory factors secreted by T and B lymphocytes, promoting the differentiation of monocytes and osteoclast precursors into osteoclasts; and (7) *Brucella* itself may also promote the differentiation and maturation of osteoclasts, although there is currently no theoretical basis to support this. MSC, mesenchymal stem cells; HSC, hematopoietic stem cell.

Bone is a dynamic tissue that is maintained by osteoblasts which are involved in bone formation, osteocytes, and osteoclasts that mediate bone resorption (Florencio-Silva et al., 2015; Manolagas, 2000). Dynamic balance of bone tissue may be disrupted by numerous factors which result in hyperosteogeny or defects, including inflammation (Deng et al., 2017; Kong et al., 2022; Przekora and Ginalska, 2017). The balance is also destroyed in *Brucella* osteoarthritis which induces bone defects, although the mechanisms that underpin these perturbations are unclear. *Brucella* grows and propagates in osteoblasts, osteocytes and osteoclasts, but it is uncertain whether the destruction of bone balance is mediated by the bacterium or by the inflammatory microenvironment caused by *Brucella* infection (Khalaf et al., 2020; Pesce Viglietti et al., 2016; Scian et al., 2012; Viglietti et al., 2019). As bone defects in *Brucella* osteoarthritis involve a variety of cell types and molecular mechanisms, this review probes the literature on bone defects in *Brucella* osteoarthritis (Giambartolomei et al., 2012; Scian et al., 2011a,b; Scian et al., 2013). We summarize and analyze research progress in the topic, identify interconnections between published studies, and determine future research directions that will enhance understanding of *Brucella* osteoarthritis (Figure 1).

## Clinical symptoms of *Brucella* osteoarthritis

Brucellosis is a zoonosis caused by the bacterium *Brucella* which typically manifests as insidious onset of fever, malaise, arthralgia, and nonspecific physical findings, including hepatomegaly, splenomegaly, and lymphadenopathy (Franco et al., 2007). Arthritis is the main symptom of brucellosis and may occur in both early and late stages of infection. However, late stage arthritis is more common with an incidence of approximately 25.6%, which is significantly higher than the other most prevalent symptoms in the late stage, fatigue (17.3%) and fever (16.4%) (Wang et al., 2022). The most usual manifestations of *Brucella* osteoarthritis are sacroiliitis, spondylitis, and peripheral arthritis (Franco et al., 2007). Approximately 6–50% of patients in all osteoarticular cases simultaneously are affected at two or more sites at the same time. Hip arthritis is the most common single-joint peripheral arthritis in the clinic in cases of single osteoarticular involvement (Bosilkovski et al., 2016). The most frequent clinical symptoms of peripheral arthritis include joint tenderness, swelling, limited movement, and joint effusion. Early imaging examination shows joint effusion and synovial thickening, and the late stage may show osteoporosis and bone destruction (Wang and Zhang, 2022).

Sacroiliac arthritis is the most common type of *Brucella* osteoarthritis with an incidence of 75.7%. Compared with other types of *Brucella* arthritis, the clinical manifestations of *Brucella* sacroiliitis are non-specific and also may coincide with spondylitis. Diagnosis depends principally on imaging methods and the specific locations of joint pain (Hashemi et al., 2007). Different clinical studies provided different descriptions of the incidence of sacroiliitis, spondylitis and peripheral arthritis. Certain studies found that the incidence of sacroiliac arthritis is the highest for *Brucella* osteoarthritis, other analyses identified that spondylitis is most frequent, whereas other studies found that the occurrence of peripheral arthritis is highest (Colmenero et al., 1991). The reason for these apparent discrepancies may be that the ages of subjects included in the studies were different as spondylitis is more likely to occur in older patients with brucellosis, whereas peripheral arthritis is more common in adolescents and children (Colmenero et al., 1991; Hashemi et al., 2007; Spernovasilis et al., 2024; Wang et al., 2022). Brucellosis spondylitis usually occurs in patients >60 years of age. The positive blood culture rate in *Brucella* osteoarthritis is low. Instead, the focus of symptoms is spondylolisthesis in the lumbar vertebrae which destabilizes the spine and which is the most important disabling bone complication of brucellosis (Alamian et al., 2021; Colmenero et al., 1991; Hashemi et al., 2007; Jiang et al., 2023; Ma et al., 2022; Spernovasilis et al., 2024; Wang et al., 2022). The clinical characteristics of brucellosis spondylitis typically include undulant fever, weakness, fatigue, lumbago, and discernible neurological impairments.

## *Brucella* and osteoarticular cells

### The multifaceted roles of osteoblasts in *Brucella* infection

*B. abortus*, *B. suis*, and *B. melitensis* infect and replicate within murine and human osteoblasts (Delpino et al., 2009). MC3T3-E1 is the most commonly used mouse cell line for studying osteoblast changes in *Brucella* osteoarthritis (Jamil et al., 2024). Following osteoblast invasion, growth and reproduction of *Brucella* affect the normal physiological state and function of osteoblasts which results in a series of metabolic impacts. *B. abortus* activates the p38 and ERK1/2 MAPK pathways to participate in the production of the chemokines MCP-1 and keratinocyte chemoattractant (KC). Activation of the p38 MAPK pathway, rather than the ERK1/2 pathway, promotes the production of matrix metalloprotein 9 (MMP-9), but neither of these pathways affects expression of the MMP-2 enzyme (Scian et al., 2012). However, the human osteoblast hFOB cell line secretes granulocyte-macrophage colony-stimulating factor (GM-CSF) in response to *Brucella* infection in a multiplicity of infection (MOI)-dependent manner which stimulates these cells to release MMP-2 (Scian et al., 2011b). *B. abortus* infection increases receptor activator of nuclear factor-κ *Β* ligand (RANKL), KC, and MMP-2 expression. Osteoblasts infected with *B. abortus* in the presence of cortisol exhibit a significant increase in both KC and MMP-2 secretion compared to untreated cells. Cortisol increases the expression of proinflammatory mediators in osteoblasts during *B. abortus* infection, and this effect can be reversed by the endogenous steroid hormone precursor dehydroepiandrosterone (DHEA) (Gentilini et al., 2018). RANKL is necessary to induce osteoclast differentiation and high expression of RANKL promotes the enhancement of bone absorption capacity.

An increase in osteoblast apoptosis destroys bone balance and stimulates osteoporosis and bone defects. Therefore, it is important to establish whether the invasion of *Brucella* into osteoblasts promotes excessive osteoblast production and abnormal levels of apoptosis. Several studies revealed that *Brucella* infection increases osteoblast apoptosis. For example, *B. abortus* infection induces osteoblast apoptosis in an MOI-dependent manner assessed by flow cytometry and TUNEL staining (Scian et al., 2012). There are no significant differences in the levels of apoptotic cells in osteoblasts infected with the *B. abortus virB10* polar mutant and uninfected control cells which suggests that osteoblast apoptosis may be related directly to the functional type four secretion system (T4SS) of *Brucella* (Scian et al., 2012; Scian et al., 2011b). Cortisol significantly increases the capacity of *B. abortus* to replicate in osteoblasts compared to untreated cells. Thus, cortisol also significantly enhances the apoptosis rate of osteoblasts infected with *Brucella*, although DHEA reverses the effect of cortisol (Gentilini et al., 2018).

**Figure 2:**
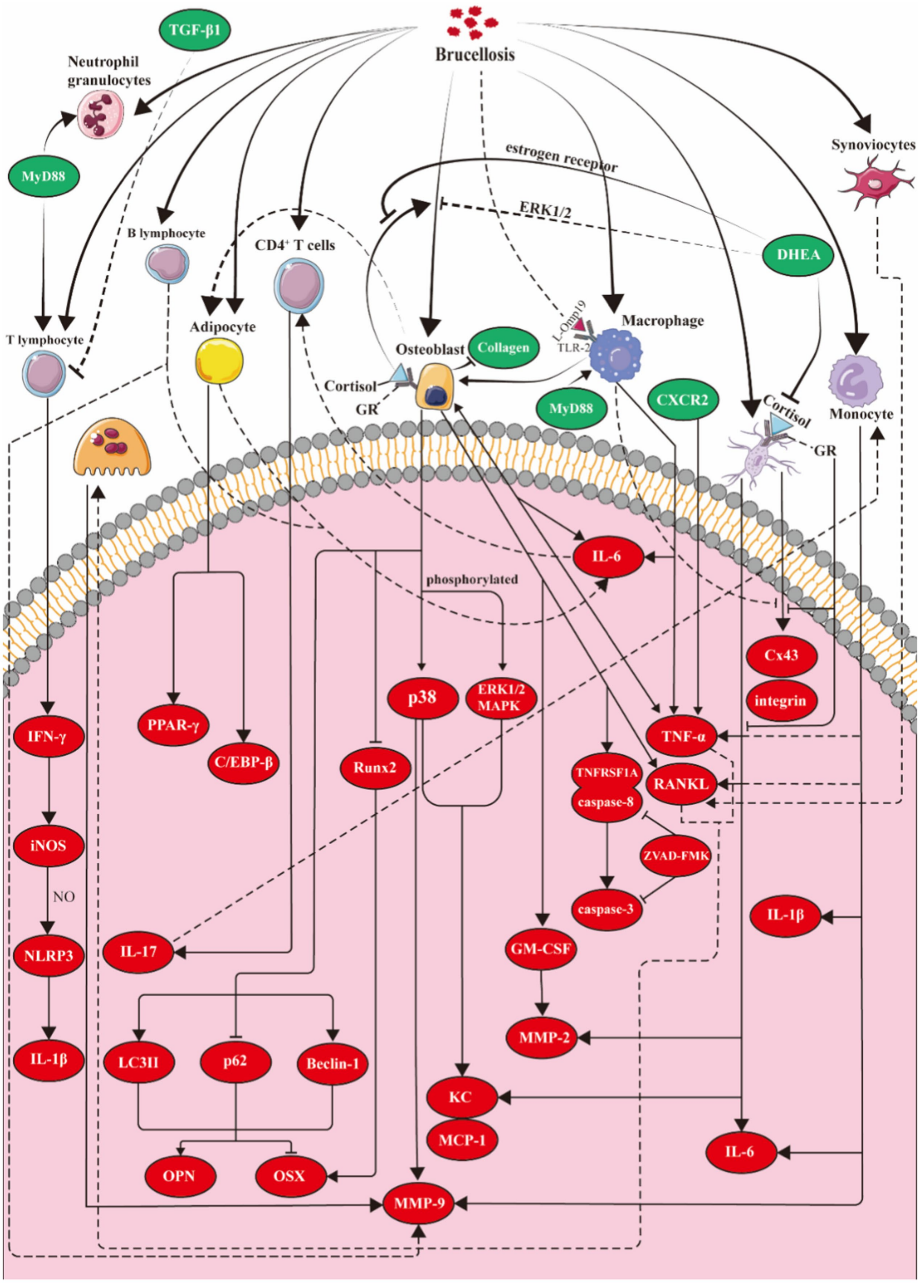
Molecular model of *Brucella* osteoarthritis. **Osteoblasts:** *Brucella* induces osteoblasts to secrete KC, MCP-1, and MMPs by activating the p38 and ERK1/2 signaling pathways. Additionally, the infected osteoblasts overexpress GM-CSF, TNF-α, and RANKL. *Brucella* also triggers apoptosis in these cells through the activation of related caspase pathways. Cortisol enhances the inhibitory effects of *Brucella* on osteoblast differentiation and function, while DHEA mitigates this suppression via estrogen receptors and the ERK1/2 pathway. Moreover, *Brucella* infection promotes autophagy in osteoblasts, regulating the expression of OPN and OSX through this autophagic process. **Osteoclasts:** TNF-α and RANKL secreted by intrinsic bone cells and infiltrating inflammatory cells promote the differentiation and maturation of osteoclasts. **Osteocytes:** After *Brucella* infection, osteoblasts overexpress IL-6, TNF-α, MMP-2, and RANKL, while suppressing the expression of Cx43. During the infection, the application of cortisol enhances these two effects in osteoblasts, whereas dehydroepiandrosterone can reverse the effects of cortisol. **Synovial cells:** Following *Brucella* infection, synovial cells upregulate anti-apoptotic factors (CIP-2, clusterin, livin, and P21/CIP/CDKN1A) to inhibit apoptosis and overexpress RANKL to promote osteoclast differentiation. **Macrophages:** Following *Brucella* infection in mice, macrophages produce pro-inflammatory cytokines such as TNF-α, IL-6, and IL-1, but do not produce RANKL or TGF-β. The production of TNF-α and IL-6 requires the involvement of MyD88 and TLR2. **T cells:** MyD88 plays a crucial role in enhancing T cell production of IFN-γ. IFN-γ induces the expression of iNOS in macrophages, leading to the production of nitric oxide (NO), which in turn inhibits the excessive activation of the NLRP3 inflammasome and reduces the production of IL-1β. **B cells:** *Brucella* infection of B lymphocytes induces the overexpression of MMP-9, RANKL, TNF-α and IL-6. **Monocytes:** The activation of TLR2 and the overexpression of TNF-α and GM-CSF stimulate monocytes to overexpress MMP-9. **Adipocytes:** Adipocytes infected with *Brucella* inhibit osteoblast mineralization by expressing IL-6. Concurrently, the infected osteoblasts stimulate adipocyte differentiation by inducing PPAR-γ and C/EBP-β. iNOS, inducible nitric oxide synthase; Cx43, gap junction protein connexin 43; DHEA, dehydroepiandrosterone; KC:CXCL1, chemokine (C-X-C motif) ligand 1; MCP-1, monocyte chemoattractant protein-1; MMPs, matrix metalloproteinases; GM-CSF, granulocyte-macrophage colony-stimulating factor; TLRs, toll-like receptors; PPARγ, peroxisome proliferator-activated receptor γ; C/EBP-β, CCAAT/enhancer binding protein β.

Osteoblast differentiation both *in vivo* and *in vitro* is characterized by deposition and mineralization of the bone matrix. The normal differentiation of osteoblasts is necessary for the secretion and mineralization of the matrix and the formation of osteocytes. *B. abortus* infection inhibits osteoblast differentiation in an MOI-dependent manner (Scian et al., 2012). Cortisol increases the inhibition of mineral and organic matrix deposition in *B. abortus*-infected osteoblasts which is neutralized by DHEA. The regulation of this process may depend on the ERK1/2 MAPK signaling pathway (Gentilini et al., 2018). *B. abortus* infection also induces autophagy in osteoblasts. Autophagy affects type I collagen (COL1A1) secretion and bone matrix calcification functions of osteoblasts, but does not impact the expression of RANKL. Moreover, *B. abortus* regulates the expression of the osteogenic osteopontin (OPN) protein and transcription factor osterix (Osx) through the autophagy pathway, but not of the runt-related transcription factor 2 (RUNX2) transcription factor that is implicated in osteoblast differentiation (Figure 2) (Viglietti et al., 2018).

### Infection of osteoclasts by *Brucella*

Since the mouse macrophage cell line RAW264.7 and bone marrow-derived macrophages (BMDM) are target cells for *Brucella*, these two types of cells are also the most commonly used in vitro models for studying brucellosis. Interestingly, BMDM can be induced to differentiate into osteoclasts by RANKL and macrophage colony-stimulating factor (M-CSF), playing a significant role in the basic research of the pathogenesis of *Brucella* osteoarthritis (Jamil et al., 2024). Osteoclasts are multinucleated bone-resorbing cells that are key players in bone remodeling for health and disease, and abundant brucellosis antigens were identified in multinucleated osteoclasts of mice with brucellosis. Extensive co-localization of *Brucella* and osteoclasts was revealed by double immunofluorescence staining of mouse tail vertebrae which indicates that the bacterium colonizes osteoclasts. However, whether osteoclasts are affected by *Brucella* infection remains to be clarified (Khalaf et al., 2019).

Elevated osteoclast activity is a major contributor to inflammatory bone loss in chronic inflammatory diseases. Dissimilar osteoclast precursor (OCP) subsets respond differently to chronic inflammation. Thus, certain OCPs react to inflammation and display enhanced osteoclastic potential, whereas other subsets remain stable and do not respond to chronic inflammation (Meirow et al., 2022). Bone defects in *Brucella* osteoarthritis are closely connected to the occurrence and development of chronic inflammation. However, equivalent studies have not yet been conducted with respect to *Brucella* osteoarthritis which warrants further exploration (Meirow et al., 2022).

*Brucella abortus* 2,308 is a highly virulent strain that affects the fusion and growth of mature osteoclasts, but does not induce osteoclast death regardless of changes in virulence. Mature osteoclasts infected with *Brucella* vaccine strains show more cell fusion compared to uninfected cells and form larger cells with more nuclei. In contrast, mature osteoclasts infected with *B. abortus* 2,308 end to decrease and the number of nuclei diminishes. However, although the fusion of mature osteoclasts is reduced after infection with *B. abortus* 2,308, no differences are apparent in bone absorption capacity and numbers of cells compared to uninfected osteoclasts. *B. abortus* 2,308 may slow down osteoblast fusion while enhancing the bone absorption capacity of osteoclasts, simply because delayed cell maturation masks this phenomenon. When Wild-type *B. abortus* 2,308 was used to infect osteoclast precursor (tartrate-resistant acid phosphatase-positive, number of nuclei <3) and the number of mature osteoclasts was smaller than in control cells, the degree of cell fusion was reduced compared to the control cells. The bone absorption capacity was also less with the wild-type strain. These results indicate an unexpected, direct, and negative effect on osteoclast growth and functional activity when infection occurs at the precursor stage. Finally, osteoblasts do not promote the formation or differentiation of osteoclasts through direct or indirect connections which is contrary to the conclusions of previous studies. Further analysis is required (Gentilini et al., 2018; Khalaf et al., 2020; Scian et al., 2012; Viglietti et al., 2018).

### *Brucella* and osteocytes

Osteocytes are the most abundant cells in bone tissue. These cells secrete numerous factors that regulate the differentiation of osteoblasts and osteoclasts during bone remodeling. *B. abortus* infection induces osteocytes to secrete MMP-2, RANKL, and KC. In addition, supernatants from *B. abortus*-infected osteocytes promote the differentiation of bone marrow-derived monocytes (BMM) into osteoclasts in the presence of tumor necrosis factor alpha (TNF-*α*) and RANKL cytokines. *B. abortus* directly invades osteocytes and inhibits the expression of the gap junction protein connexin 43 (Cx43), but does not induce apoptosis. However, culture supernatants of *B. abortus*-infected macrophages concurrently suppress the expression of Cx43 and integrin transmembrane receptor in osteocytes, thereby inducing significant apoptosis and subsequently triggering impaired bone integrity (Pesce Viglietti et al., 2016).

Hormones regulate the bone remodeling process. Cortisol and DHEA also modulate *Brucella* infection of bone cells. Cortisol treatment inhibits the expression of interleukin-6 (IL-6), TNF-α, MMP-2, and RANKL in *B. abortus*-infected osteocytes, whereas DHEA reverses the inhibitory effect of cortisol on MMP-2 production. As mentioned above, the culture supernatant of *Brucella*-infected osteocytes contains large amounts of TNF-α and RANKL that induce the differentiation of BMMs into osteoclasts. However, as cortisol blocks the secretion of TNF-α and RANKL by osteocytes, the hormone inhibits the occurrence of osteoclasts partly because cortisol increases expression of osteoprotegerin (OPG) which is a natural antagonist of RANKL that partially impedes osteoclast differentiation. *B. abortus* infection inhibits Cx43 expression, expression increases when cortisol is added during infection. To play this role, cortisol binds to glucocorticoid receptor (GR) which is expressed in bone cells. But DHEA treatment partially reverses this effect of cortisol. The sensitivity of cells to cortisol depends not only on the serum concentration of cortisol, but also on the ratios between different GR subtypes. GRb does not bind to glucocorticoids and inhibits GRa-mediated transcriptional activation. The expression of both GRa and GRb is reduced in *B. abortus-*infected osteocytes, but GRb expression decreases more. Therefore, the GRa/GRb transcriptional ratio increases, and the sensitivity to cortisol is enhanced. Simultaneously, intracellular cortisol levels affect cell function. The levels of cortisol are also dependent on the ratio between the isoenzyme 11β-steroid dehydrogenase type 1 (11β-HSD1), which converts cortisone to cortisol, and type 2 (11β-HSD2), which converts cortisol back to cortisone. In osteoblasts infected with *Brucella*, there is an increased expression of 11β-HSD1 and a decreased expression of 11β-HSD2, leading to an elevated 11β-HSD1/11β-HSD2 ratio. This promotes the conversion of intracellular cortisone to cortisol, thereby enhancing the effects of cortisol (Figure 2) (Viglietti et al., 2019).

### Effects of *Brucella* infection on fibroblast-like synoviocytes

Fibroblast-like synoviocytes exert a pivotal role in the pathogenesis of inflammatory arthritis by producing MMPs that degrade collagen, as well as cytokines and chemokines that mediate leukocyte recruitment and activation. *Brucella* invades human synovial cells which leads to intracellular replication of the bacterium. Infection with *Brucella* induces high expression of MMP-2 and pro-inflammatory mediators, including IL-6, IL-8, MCP-1, and granulocyte-macrophage colony-stimulating factor (GM-CSF) in synovial cells. The upregulation of MMP-2 and these pro-inflammatory mediators is mediated through toll-like receptor 2 (TLR2) recognition of *Brucella* antigens which is independent of bacterial viability. Additionally, culture supernatant from *Brucella*-infected synovial cells induces the migration of monocytes and neutrophils while stimulating secretion of MMP-9 by these cells. This effect may be attributed to the presence of GM-CSF and IL-6 in the supernatant. Thus, bone damage in *Brucella* osteoarthritis may result from the production of MMPs and pro-inflammatory mediators by synovial cells, along with phagocytes recruited to infected lesions (Scian et al., 2011a).

*Brucella abortus* infection inhibits synovial cell apoptosis by upregulating anti-apoptotic factors including cellular inhibitor of apoptosis 2 (cIAP2), clusterin, livin, and P21/CIP/CDNK1A, but infection does not affect the expression of apoptotic proteins such as bcl2 associated agonist of cell death (Bad), bcl2 associated x (Bax), cleaved procaspase 3, cytochrome c oxidase (CytC), and TNF-related apoptosis-inducing ligand (TRAIL). In addition, infection induces synovial cells to overexpress the adhesion molecules CD54 and CD106 which results in enhanced adhesion of monocytes and neutrophils to synovial cells. However, despite increased adhesion, *Brucella* infection inhibits monocyte-and neutrophil-induced synovial cell apoptosis. Finally, *B. abortus* infection provokes the expression of RANKL by synovial cells and stimulates peripheral monocytes to differentiate into osteoclasts which may lead to osteoporosis and bone defects in osteoarthritis (Figure 2) (Scian et al., 2013).

## Brucellosis and immune cells

*Brucella* possesses various virulence factors, such as lipopolysaccharides, Type IV Secretion System and BvrR/BvrS, and the outer membrane proteins (Omps). These virulence factors suppress the immune response by interfering with the host’s innate and adaptive immune recognition and responses, thereby promoting *Brucella* replication and sustaining infection in the host. During this process, the chronically existing inflammatory microenvironment may partially impact the normal physiological functions of bone tissue cells, ultimately leading to bone deficits in patients with *Brucella* osteoarthritis (Elrashedy et al., 2022).

### The role of macrophages in *Brucella* osteoarthritis

*Brucella* virulence relies mainly on the ability to invade and replicate within professional and nonprofessional phagocytes, among which macrophages are the major target cells in infected mammals (Li et al., 2017). Osteoclasts are differentiated *in vitro* from myeloid cells such as bone marrow macrophages (Boyle et al., 2003; Tsai et al., 2023). A consensus has emerged in osteoimmunology that a close relationship exists between the immune system and bone, and that macrophages are a key feature of the bone immune system (Coury et al., 2019). As highlighted above, macrophages also are important nodes in the pathogenesis of *Brucella* osteoarthritis (Figure 1).

*Brucella abortus* infection induces macrophages to overexpress TNF-*α* through the TLR2 signaling pathways. TNF-α stimulates the differentiation of BMMs into osteoclasts. This cascade of cytokine secretion and the accompanying physiological and pathological alterations are dependent on the Omp19 outer membrane lipoprotein of *B. abortus*, although Omp19 is not essential for survival of the bacterium. Activation of IL-1, macrophage colony stimulating factor receptor (c-Fms), TNF-α, prostaglandin E2 (PGE2), and transforming growth factor *β* (TGF-β) receptors on osteoclast surfaces enhances osteoclast production *in vitro* and bone resorption *in vivo*. TNF-α may be involved in tumor necrosis factor receptor 1 (TNF-R1) signaling through tumor necrosis factor receptor-associated factor-6 (TRAF6) and activation of these receptors may exert a synergistic effect on RANK-mediated TRAF6 activation (Delpino et al., 2012). RANK is an osteoclast cell-surface receptor that binds to RANKL.

Other studies also revealed that macrophages affect osteoclast differentiation via TNF-α, thereby promoting osteoporosis and defects in patients with *Brucella* osteoarthritis. The specific mechanism involves *B. abortus*-mediated induction of expression of IL-6 by infected macrophages so that IL-6 is present in enhanced concentrations in the surrounding medium. Stimulation of CD4(+) T cells by the supernatant induces these cells to secrete IL-17 which promotes the expression of TNF-α in OCPs. This eventually leads to accelerated osteoclast differentiation and stimulates bone erosion (Figure 2) (Giambartolomei et al., 2012).

### T cell immunity and brucellosis

As highlighted above, *Brucella*-infected macrophage culture supernatant stimulates CD4(+) T cells to secrete RANKL and IL-17 to induce osteoclast differentiation. These effects are weakened or disappear after treatment with IL-17A–blocking antibody (Giambartolomei et al., 2012). Thus, T cells induce osteoclast differentiation mainly through IL-17 overexpression. In fact, T cells overexpress both RANKL and IL-17 after treatment with the supernatant, but RANKL does not accelerate osteoclast differentiation when the expression of IL-17 is inhibited. RANKL and M-CSF are well-characterized inducers for differentiation of monocytes into osteoclasts. Although T cells may express RANKL, the production of the protein is not the main reason for osteoclast generation compared with mesenchymal cells such as osteoblasts. Moreover, T cells activated by *Brucella*-infected macrophage culture supernatant express RANKL, IL-17, and IL-10. IL-10 inhibits the RANKL/RANK interaction, thereby preventing RANKL-induced osteoclast generation. Further exploration of the roles of RANKL, IL-17, and IL-10 in *Brucella*-mediated T cell activation is required.

T cell antigen receptor *ζ* chains are downregulated and T cell function is impaired when T cells are exposed continuously to antigens and chronic systemic inflammation. These effects may be a physiological reaction to dampen the long-standing immune response. However, this process not only reduces the immune response, but also impairs the killing of foreign bodies and pathogens which results in the persistence of disease. Chronic brucellosis is a long-term infectious disease that may involve impaired T-cell function (Bronstein-Sitton et al., 2003). Live attenuated *Brucella* sheep vaccine administered orally activates systemic and mucosal type I helper T cells Th1 and Th17 and induces the latter to produce IL-17 and IL-22, thereby exerting a protective effect on respiratory transmission of *B. melitensis* 16M (Clapp et al., 2011). TGF-β1 levels in patients with chronic brucellosis are significantly higher than uninfected patients. Moreover, TGF-β1 expression in peripheral blood mononuclear cells stimulated by the *Brucella* antigen increases by a factor of two *in vitro*, and the differentiation of these cells into T lymphocytes is weakened. In addition, TGF-β1 expression is downregulated and T lymphocyte proliferation is enhanced when a TGF-β1 neutralizing antibody is used to treat peripheral blood mononuclear cells after stimulation with *Brucella* antigen (Elfaki and Al-Hokail, 2009). These observations demonstrate the close relationship between T cell immunity and brucellosis and may also reflect the chronicity of brucellosis and osteoarthritis (Figure 2).

### Do B cells have a role in *Brucella* osteoarthritis?

B cells are adaptive immune cells that produce specific immunoglobulins which bind to pathogens. These cells also may be involved in the innate immune response (Tsay and Zouali, 2018). *B. abortus* infection activates and prolongs the survival time of B cells (Goenka et al., 2011). Thus, B lymphocytes provide an infection niche for intracellular *B. abortus* (Goenka et al., 2012). B cells promote communication with osteoclasts and osteoblasts through diverse cytokines and participate in the development of osteoporosis (Frase et al., 2023). As brucellosis involves both inflammation and bone tissue, B cells may play a role in *Brucella* osteoarthritis. However, only one study to date has described a relationship between B cells and bone erosion in *Brucella*-mediated osteoarthritis. *B. abortus* that directly infected B cells induced B cells to overexpress MMP-9, RANKL, TNF-*α*, and IL-6. In addition, the culture supernatant of these cells induced differentiation of BMM into osteoclasts. This effect was inhibited by OPG, a decoy receptor for RANKL, which indicates that RANKL plays a major differentiation role in this supernatant (Figure 2) (Viglietti et al., 2016).

### Monocytes as intracellular hosts for *Brucella*

Monocytes derived from bone marrow are the largest blood cells and form a crucial branch of the bodily defense system (Austermann et al., 2022). Monocytes exert homeostatic functions and differentiate into tissue macrophages in the steady state. Monocytes are recruited to the site of inflammation and ultimately differentiate into inflammatory macrophages or dendritic cells (Guilliams et al., 2018). Monocytes are the sole source of osteoclasts (Boyle et al., 2003; Tsai et al., 2023). *B. abortus* persists in bone marrow where the bacterium infects monocytes, neutrophils, and granulocyte-monocyte-progenitor cells, among which monocytes are the most probable hosts for *Brucella* in the bone marrow and may be the source of the frequent relapses observed in antibiotic-treated individuals (Gutiérrez-Jiménez et al., 2018).

As outlined above, the invasion of osteoblasts by *Brucella* increases the expression of MCP-1 which induces the migration of the THP-1 human monocyte cell line to the lesion site. Infection with *B. abortus* induces MMP-9 secretion from monocytes. This secretion is activated by whole, heat-killed *B. abortus* as well as by Omp-19 derived from *B. abortus*. Therefore, the overexpression of MMP-9 is promoted by the structural protein Omp-19 in *B. abortus*. These effects are mediated by TLR2 and by the action of TNF-α produced by monocytes. Osteoblasts secrete MMP-9, monocyte Chemotactic Protein-1 (MCP-1), and GM-CSF during invasion by *B. abortus*. The MCP-1 chemokine induces monocyte aggregation whereas GM-CSF stimulates secretion of TNF-α by monocytes which in turn stimulates the secretion of MMP-2 and MMP-9 by osteoblasts, thereby forming a cycle of secretion. This process eventually leads to breakdown of the organic bone matrix (Scian et al., 2011b). Culture supernatants from *Brucella*-infected osteoblasts induce monocytes to produce TNF-α, IL-8, IL-1*β*, and IL-6 through GM-CSF. Furthermore, infected osteoblasts produce low levels of IL-8 and MCP-1 chemokines but do not generate proinflammatory cytokines IL-1β, IL-6, and TNF-α. However, the chemokines produced by osteoblasts were substantially increased and IL-6 was produced when osteoblasts were stimulated with culture supernatants from *Brucella*-infected human monocytes which was not observed by brucellosis infection alone (Figures 1, 2) (Delpino et al., 2009).

## Special cell types implicated in *Brucella* osteoarthritis

### *Brucella abortus* and adipocyte differentiation

Osteoblasts and adipocytes crosstalk as both cell types originate from mesenchymal stem cells, and the tendency of mesenchymal stem cells to differentiate into adipocytes or osteoblasts is a potential factor in bone loss. Additionally, osteoblasts may transform into adipocytes and vice versa in response to changes in the surrounding microenvironment. Pro-inflammatory cytokine IL-6 in the culture supernatant of *B. abortus*-infected adipocytes inhibits the deposition of the osteoblast mineral matrix and decreases the transcription of RUNX2 that is necessary for osteoblast differentiation, but does not affect secretion of the osteoblast organic matrix or the expression of RANKL (Freiberger et al., 2023). Moreover, osteoblasts infected with *B. abortus* stimulate adipocyte differentiation by inducing peroxisome proliferator-activated receptor *γ* (PPAR-γ) and CCAAT enhancer-binding protein β (C/EBP-β) which indicates that a closed loop is formed between osteoblasts and adipocytes. When *B. abortus* invades these cells, osteoblasts promote adipocyte differentiation which in turn increases overexpression of IL-6. This overproduction accelerates the inhibition of inorganic mineral deposition in osteoblasts leading to a cycle that culminates in osteoporosis and bone defects (Figures 1, 2) (Freiberger et al., 2023).

### Involvement of myeloid-derived suppressor cells in the pathology of *Brucella* osteoarthritis

*Brucella* persists in the bone marrow of infected individuals for sustained periods. Myelopoiesis in response to pathogenic stimuli is a fundamental mechanism that protects the host from blood cell perturbations. Myelopoiesis largely manifests as an expansion of activated neutrophils and monocytes. Chronic infections or cancer induce long-term myelopoiesis with low-intensity continuous stimulation. Although myeloid cells produced under these conditions are similar to neutrophils and monocytes in both morphology and phenotype, these cells have different genomic and biochemical characteristics and functional activities. The cells are denoted bone marrow-derived suppressor cells because of a strong ability to inhibit all types of immune response (Ezernitchi et al., 2006; Gabrilovich, 2017; Zhuang et al., 2012).

Mononuclear bone marrow-derived suppressor cells differentiate into osteoclasts and play a vital role in osteoarthritis. For example, enhanced osteoclast bone absorption in patients with rheumatoid arthritis leads to periarticular erosion and systemic osteoporosis (Charles et al., 2012). Analysis of the bone marrow cell population showed that Ly6C^hi^CD11b^−/lo^ cells increased in patients with rheumatoid arthritis. *In vivo* experiments revealed that transplantation of these cells increased arthritis inflammation in animal models. Subsequent studies revealed that Ly6C^hi^CD11b^−/lo^ cells inhibit the proliferation of CD4^+^ and CD8^+^T cells by producing NO, similarly to monocyte myeloid suppressor cells. Thus, the proliferation of the bone marrow myeloid precursor population with osteoclastic and T cell–suppressive activity is key to inflammatory arthritis and the resulting bone damage (Charles et al., 2012). Analogously, there is a large increase during chronic inflammation of OCPs with myeloid-derived suppressor cells properties and differentiation into osteoclasts for bone erosion. However, the molecular markers on the surfaces of OCP populations differ from those described above. Two distinct OCP subsets were identified: Ly6C^hi^CD11b^hi^ inflammatory OCPs (iOCPs) induced during chronic inflammation and homeostatic Ly6C^hi^CD11b^lo^ OCPs (hOCPs) which remained unchanged. The hOCP subset was consistent with surface molecular markers of the bone marrow cell population described previously which indicates the same cell population. However, these hOCPs did not respond to the inflammatory environment whereas iOCPs, which usually display low osteoclastogenesis potential, were significantly amplified in the inflammatory microenvironment, and expressed higher levels of resorptive and metabolic proteins (Cremers et al., 2017).

Further studies have shown that TNF-*α* and the alarmin S100A8/A9 regulate iOCP activity during chronic inflammation (Cremers et al., 2017; Meirow et al., 2022; Sade-Feldman et al., 2013). Mouse monocytes were divided into classical Ly6C^high^ and non-classical Ly6C^low^ monocyte subsets using Ly6C^high^ as a surface molecular marker of amplified monocytes. The Ly6C^high^ monocyte subsets in the mouse model of osteoarthritis were significantly larger than those in the negative control group which suggests that Ly6C^high^ monocytes, rather than Ly6C^low^ monocytes, are responsible for primary osteoclast differentiation in arthritis. However, with respect to the molecular marker CD11b on the cell surface, it is believed that differentiation of CD11b^+^ monocytes into osteoclasts is intensified in the inflammatory environment (Ammari et al., 2018). Human CD16^+^ and CD14 + monocytes are equivalent to mouse Ly6C^low^ and Ly6C^high^ monocytes, respectively. A positive association exists between the proportion of peripheral blood OCPs, defined as CD3^−^CD19^−^CD56^−^CD11b^+^CD14^+^, and the disease activity score in follow-up samples from patients with psoriatic arthritis receiving anti-TNF therapy (Sucur et al., 2014). These observations confirm that the proliferation of the bone marrow myeloid precursor population with osteoclastic and T cell–suppressive activity is key to inflammatory arthritis and the resulting bone damage. Patients with *Brucella* osteoarthritis have been in a state of chronic infection for a sustained period, and the infection may be accompanied by obvious bone erosion and inhibition of T cell function. Myeloid-derived suppressor cells may play a significant role in the pathology of *Brucella* osteoarthritis although further studies are required.

## Special inflammatory factors in *Brucella* osteoarthritis

The roles of common inflammatory factors, including TNF-α, IL-1β, IL-6, and IL-10, in *Brucella* osteoarthritis have been referred to above and also have been described in detail elsewhere. Here, we outline the role of relatively rare inflammatory factors, myeloid differentiation primary response 88 (MyD88), C-X-C chemokine receptor type 2 (CXCR2), and interferon-*γ* (IFN-γ), in the condition.

### MyD88

MyD88 is an adaptor protein that plays a critical role in transmitting signals from TLRs, IL-1R, and IL-18R (Adachi et al., 1998; Miller et al., 2006). Upon activation, MyD88 initiates cytokine production through TLRs (Kawai and Akira, 2005) in a signaling pathway that is associated with the development of arthritis and osteoclastogenesis in murine models of bacterium-induced focal complications (Chen et al., 2015; Joosten et al., 2003; Kyo et al., 2005). *Brucella* may be recognized by host cells through diverse TLRs, including TLR2, TLR4, TLR6, and TLR9 (Campos et al., 2004; de Almeida et al., 2013; Ferrero et al., 2014; Gomes et al., 2016; Vieira et al., 2013).

Ankle swelling of MyD88^−/−^ mice with *Brucella* osteoarthritis was less than in wild-type mice in the early stages of infection, but swelling in the mutant mice was elevated compared with wild-type animals after 7 days. In parallel, the fraction of interferon-producing CD4^+^ and CD8^+^T cells in MyD88^−/−^ mice was less than in wild-type mice on days 7 and 14 following infection. By day 28 post-infection, the levels of IFN-*γ* production by T cells were similar in both mouse strains. These observations indicate that MyD88 stimulates the inflammatory response in the early stage of infection to efficiently eliminate *Brucella* and promote rapid regression of inflammation which may be related to the expression of IFN-γ induced by MyD88 (Figure 2) (Lacey et al., 2017).

### CXCR2

Chemokines are a class of secreted small cytokines or signaling proteins with the ability to induce directed chemotaxis in nearby reactive cells. Chemokine receptors are G protein-coupled receptors that harbor seven transmembrane domains located on the surfaces of white blood cells and which transmit signals intracellularly. Nineteen chemokine receptors have been identified to date and are grouped into four families based on the type of chemokine bound by the receptor: CXCR binds to CXC chemokines, CCR recognizes CC chemokines, CX3CR1 binds to CX3C chemokines (CX3CL1), and XCR1 interacts with both XCL1 and XCL2 chemokines.

Chemokines CXCL2, CCL2, and CCL3 in IFN-*γ*^−/−^ mice are upregulated on days 23 and 30 after *Brucella* infection, whereas CXCL1 is upregulated on day 30 compared with wild-type mice.^73^ Wild-type and CCR2^−/−^ mice treated with anti-IFN-*γ* and intraperitoneally infected with *B. melitensis* were monitored for morbidity, clinical scores, tissue bacterial load, and swelling levels which revealed that CCR2 is not a critical mediator of *Brucella*-induced focal inflammation. CXCL1 and CXCL2 both are CXCR2 ligands and are upregulated in brucellosis-type osteoarthritis. The role of CXCR2 in *Brucella*-induced inflammation was analyzed further in CXCR2 knockout mice which showed delayed onset, lower clinical scores, and reduced swelling compared with wild-type animals. Concurrently, IFN-γ^−/−^/CXCR2^−/−^ mice had less paw and tail swelling and less osteomyelitis than IFN-γ^−/−^ mice infected with *B. melitensis*. Neutrophil infiltration was seen in both cases, although neutrophil recruitment in the inflammatory tissue of IFN-γ^−/−^/CXCR2^−/−^ mice was reduced by approximately 40% compared to IFN-γ^−/−^ mice. Thus, inhibition of CXCR2 expression helps to control tissue inflammation, reduces tissue neutrophil infiltration, and alleviates clinical symptoms in *Brucella* osteoarthritis (Lacey et al., 2016).

### IFN-γ

The IFN-γ cytokine is produced only by activated T cells, natural killer (NK) cells, and NKT cells. The protein has antiviral, immunomodulatory, and anti-tumor properties. IFN-γ activates antigen-presenting cells and promotes differentiation of Th1 cells by upregulating transcription factor T-box transcription factor (TBX21). IFN-γ is a signature cytokine in Th1 cells, although NK and CD8^+^T cells also produce the cytokine.

IFN-γ plays an important role in brucellosis and most likely is associated with the chronicity of the condition (Khatun et al., 2021). Wild-type mice have natural resistance to *Brucella* and chronic brucellosis, such as brucellosis meningitis and *Brucella* osteoarthritis, cannot be induced in these animals. Therefore, it is impossible to produce a suitable murine chronic brucellosis model in wild-type animals. However, mice lacking IFN-*γ* develop arthritis regardless of the route of *Brucella* infection (Lacey et al., 2016). For example, IFN-γ in (+874 A/T in intron 1) TT and + 5,644 T/A TT genotypes, which reportedly are associated with high IFN-γ production, are linked to brucellosis susceptibility in Iranian subjects (Eskandari-Nasab et al., 2013). Furthermore, IFN-γ levels in chronic brucellosis patients are lower than in acute brucellosis patients (Ghaznavi Rad et al., 2017). Lipoproteins of *B. abortus* inhibit increased IFN-*γ* expression and activation of interferon regulatory factor 1 (IRF-1) through IL-6. IRF-1 is a critical protein for induction of class II major histocompatibility complex transactivator (CIITA) and thereby contributes to the downregulation of CIITA mRNA transcription. This downregulation results in reduced major histocompatibility complex class II (MHC-II) surface expression on human monocytes with a consequent decrease in antigen presentation to CD4^+^ T cells. This reduction in *Brucella* antigen presentation and a weakened immune response promote chronic *Brucella* infection (Velásquez et al., 2017). Mice that lack adaptive immune cells and innate lymphoid cells (ILCs) develop arthritis, neurological complications, and meningitis after *Brucella* infection of the lungs. Transcriptomic analysis of infected brain tissue revealed significant upregulation of genes associated with inflammation and the interferon response. Type II interferon deficiency led to *Brucella* colonization of the mouse brain, whereas deficiency of both type I and type II interferons resulted in a higher intracerebral brucellosis load in the brain and faster onset of clinical symptoms of neurobrucellosis (Moley et al., 2023). Several mechanisms by which IFN-*γ* inhibits *Brucella*-induced arthritis have been reported. The lack of IFN-γ leads to a decrease in the expression of nitric oxide synthase and a corresponding decrease in the production of nitric oxide. Nitric oxide directly inhibits secretion of IL-1β from brucellosis-infected macrophages and blocks activation of the inflammatory body nucleotide-binding oligomerization domain, leucine-rich repeat and pyrin domain-containing 3 (NLRP3) *in vitro*. It has also been reported that nitric oxide kills intracellular *Brucella* and reduces bacterial load. In conclusion, IFN-*γ* restriction in the development of *Brucella* osteoarthritis may be achieved by inducing nitric oxide to curb the release of inflammatory cytokines, inhibit the excessive activation of inflammasomes, and kill some intracellular bacteria (Figure 2) (Campos et al., 2019; Gomes et al., 2021; Lacey et al., 2019).

## Laboratory animal models for brucellosis research

The pathogenesis of *Brucella* osteoarthritis is intricate and many aspects of the disease remain under-investigated which necessitates further *in vivo* and *in vitro* studies. Animal models serve as crucial tools for pathogenicity research and the development of an appropriate animal model is paramount for disease understanding. Establishing suitable animal models for brucellosis is a considerable challenge as the condition is multifaceted. Nevertheless, mice are employed extensively as animal models to investigate chronic *Brucella* infections which enables dissection of specific causative factors, characterization of host immune responses, and effective evaluation of treatments and vaccines. Additionally, rats, guinea pigs, and monkeys have been utilized as models for brucellosis investigation. Here, we focus primarily on animal models that are applicable for exploring brucellosis-induced osteoarthritis (Silva et al., 2011).

### Murine models

Although the study of *Brucella* chronic infectious diseases using mice is imperfect, environmental, economical, convenience and time factors ensure that mice are the most commonly used animal model for the condition. Strain-specific differences are observed in *Brucella* infection in mice. For example, BALB/c mice are more susceptible to infection with virulent strains of *B. abortus* than C57BL/6 or C57BL/10 strains. However, clinical symptoms and time of death were the same in BALB/c and C57BL/6 mice with IFN-*γ* gene knockouts, so both strains of mice may be used to create models of *Brucella* osteoarthritis (Murphy et al., 2001). Thus, most studies of *Brucella* osteoarthritis use C57BL/6, C57BL/10, or BABL/c knockout backgrounds. For example, a virulent bioluminescent strain of *B. melitensis* was intraperitoneally injected into BALB/c mice to identify the location of colonization in mice and to evaluate dispersion of the bacteria. An initial rapid spread of bacteria throughout the mouse was observed using *in vivo* bioluminescence imaging, followed by a distinct luminescence pattern in the tail that correlated with the anatomical arrangement of the vertebrae. These observations suggest the potential for direct colonization of osteoarticular tissues (Magnani et al., 2013).

Knockout C57BL/6 mice with mutations Rag1^−/−^, IFN-γ^−/−^, Rag2^−/−^, Rorc^−/−^, Tbx21^−/−^, IFN-γr1^−/−^, IFN-γr1^−/−^/Ifnar1^−/−^, or Tcra^−/−^ were used to study the role of ILCs and IFN-γ in *Brucella* osteoarthritis and neurological complications. For example, ILC-deficient Rag2^−/−^/Il2rγ^−/−^ mice were used to investigate the role of ILCs in *Brucella* invasion of the respiratory tract. The deletion of both Rag2 and X-linked Il2rγ genes leads to a lack of T cells, B cells, and all innate lymphoid cells, including NK cells, ILC1s, ILC2s, and ILC3s. These mice began to show signs of arthritis on day 27 following pulmonary infection and 90% of the animals developed arthritis within the following 20 days. In contrast, Rag2^−/−^ mice did not exhibit signs of arthritis during infection. Peak arthritis clinical scores were also higher in Rag2^−/−^/Il2rγ ^−/−^ mice than in Rag2^−/−^ mice (Moley et al., 2023). The Rag2^−/−^/Il2rγ^−/−^ mice are highly similar to NOD-scid IL2rγ^null^ (NSG), both of which are immunodeficient strains. NSG mice were employed to explore the use of NSG mice to study *Brucella* osteoarthritis and to assess the potential use of this immunodeficient mouse as a safe live attenuated vaccine. These NSG mice inoculated with *B. abortus* had decreased body temperature, reduced body weight, enlarged spleens, and deformed tails. Pathological sections showed severe inflammatory reactions in tail lesions with a large number of aggregated neutrophils, macrophages, and osteoclasts accompanied by significant bone destruction. These histopathological changes are similar to those typically observed in patients with brucellosis. These findings suggest that NSG mouse models may be used to evaluate vaccine safety and explore osteoarticular brucellosis (Khalaf et al., 2019).

Wild-type mice systemically infected with *Brucella* typically do not exhibit arthritis, but mice lacking IFN-γ develop the condition regardless of the route of infection. Therefore, IFN-γ-deficient mice are used widely as a model for the study of chronic brucellosis, including osteoarthritis and meningitis (Lacey et al., 2019; Moley et al., 2023; Murphy et al., 2001). *Brucella* replication generally is restricted to the spleen and liver and to a lesser extent to lymph nodes in immunocompetent mice, thereby limiting use of these mutant mice for studying focal inflammation that often is found in brucellosis. As chronic brucellosis is characterized by focal inflammatory symptoms, including *Brucella* osteoarthritis and neurobrucellosis, it is important to develop an animal model in which brucellosis causes these symptoms. IL-1R^−/−^ mice are more susceptible to systemic *Brucella* infection after IFN-γ knockdown, but IL-1R^−/−^ mice are more resistant to focal inflammation than wild-type mice with IFN-γ knockdown alone. Thus, mice with knocked down IFN-γ were more likely to develop symptoms such as osteoarthritis and neuritis than IL-1R^−/−^/IFN-γ^−/−^mice. Studies also have been conducted to design animal models with respect to IFN-γ regulation, including IRF-1^−/−^ mice. The IRF-1 protein is a key member of the interferon regulatory factor family. The main function of IRF-1 is to bind to interferon-stimulated response elements in target gene promoter regions, thereby exerting an important role in the interferon-induced signaling pathway. IRF-1^−/−^ mice develop acute infections in many tissues and *Brucella* replicates in the salivary glands of IRF-1^−/−^ mice. In addition, IRF-1^−/−^ mice infected with *B. abortus* develop acute hepatitis similarly to humans. Unlike in the natural hosts, IRF-1^−/−^ mice cannot control infection and die within a short period of time. However, chronic brucellosis localization in the mouse tail vertebrae was achieved when sublethal amounts of *Brucella* were used to infect IRF-1^−/−^ mice which may be helpful in establishing a brucellosis-type osteoarthritis model (Rajashekara et al., 2005).

**Figure 3:**
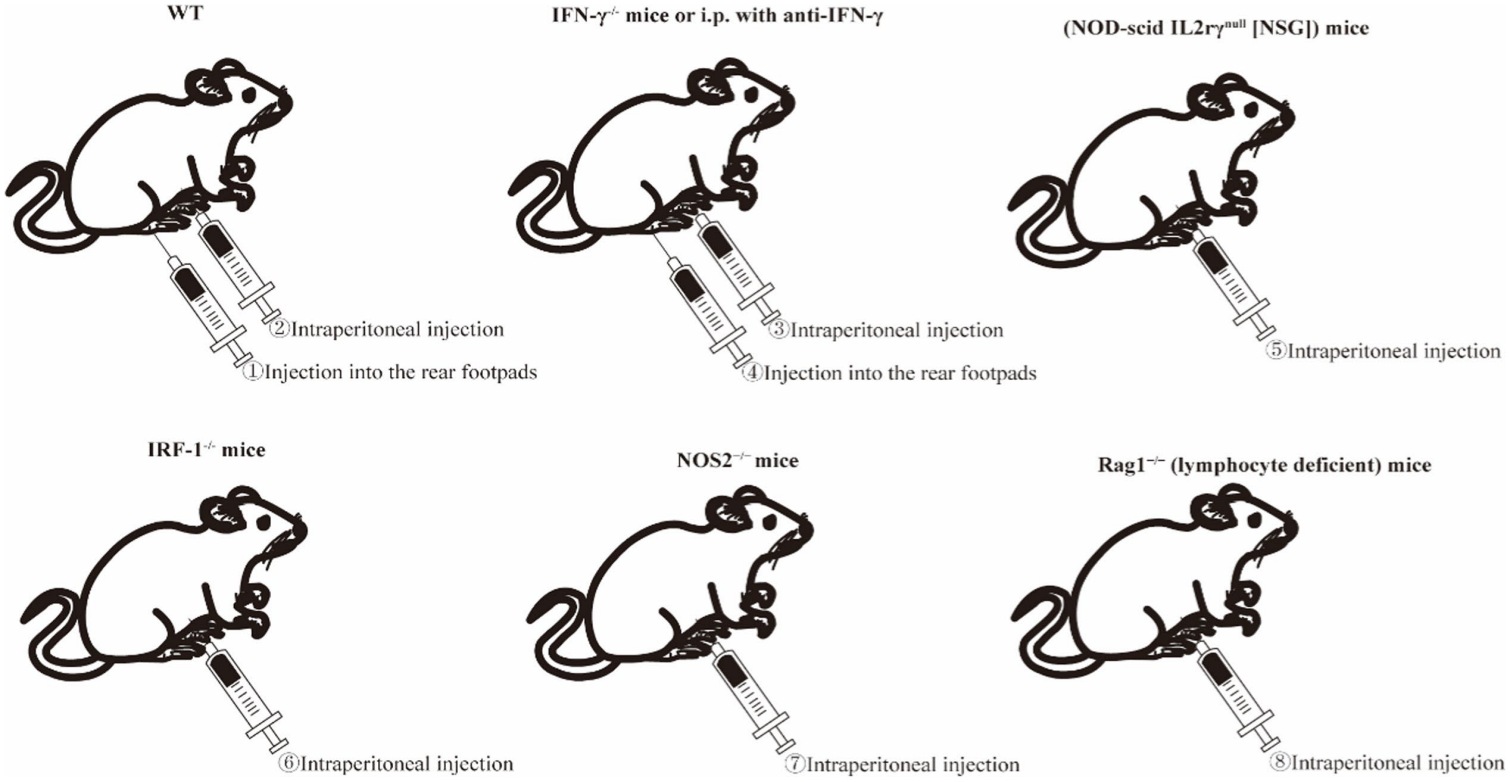
Schematic diagram of *Brucella*-induced osteoarticular inflammation model in mice. Among these, ①, ②, ③, ④, ⑤, and ⑥ represent the currently established mouse models of *Brucella*-induced osteoarticular inflammation, while ⑦ and ⑧ are potential modeling methods identified from the literature. IRF-1, interferon regulatory factor 1; NOS2, nitric oxide synthase 2; Rag1, recombination activating-1.

The main current problem with animal models for *Brucella* osteoarthritis is that, although various knockout mice models may be established successfully, part of the immune function of the mutant mice is lost. Therefore, these models do not completely simulate the pathophysiological mechanism of human *Brucella* osteoarthritis. However, attempts also have been made to build *Brucella* osteoarthritis models with wild-type mice. For example, 1 × 10^6^ CFU of *B. melitensis* 16 M were used to infect the rear footpads of C57BL/6 wild-type mice. Some footpad-infected mice demonstrated symptoms of arthritis and inflammation in the musculoskeletal system affecting the joints, as well as the surrounding muscles and soft tissues. Peak manifestations of joint arthritis and musculoskeletal inflammation were observed between days 3 and 14, and were characterized by the presence of extensive and severe combined areas of inflammation that included neutrophils, macrophages, and fibrin within the joint cavity. The first advantage of this model is that it minimizes the effect of partial gene knockouts on the overall pathogenic process. Second, *Brucella* may be transferred directly to the joints and surrounding tissues to synchronize the time of infection and inflammation. A disadvantage of this system is that *Brucella* customization into the joint is missing and it is difficult to determine whether alterations in joint inflammation are due to local immune responses or are a consequence of a change in the dissemination of *Brucella* to the joint (Figure 3) (Lacey et al., 2017).

### Non-murine laboratory models for *Brucella* osteoarthritis

As highlighted above, the breadth of animal models for *Brucella* osteoarthritis is relatively limited. For example, brucellosis spondylitis cannot be studied in the recently developed *Galleria mellonella* invertebrate (moth) model (Wang et al., 2023). The recently developed chick embryo model of *Brucella* infection offers several advantages over mouse models, including sterility, ease of handling, multiple inoculation routes, lower cost, and the absence of ethical review requirements. However, the manuscript does not describe the specific changes of bone tissue in chick embryos, and it remains unclear whether the pathological mechanisms of bone tissue in chick embryos are applicable to mammals. Therefore, the applicability of the chick embryo infection model in *Brucella* osteoarthritis research still requires further validation (Wareth et al., 2015). Vertebrates animals other than mice, including rats, guinea pigs, rabbits, dogs, goats, and monkeys, have been tested as models for brucellosis. However, due to the unique nature of brucellosis-induced osteoarthritis, it is necessary to increase bacterial inocula, modify infection methods, and extend incubation periods when developing animal models that encompass pathophysiological reactions in the spinal joints. Consequently, establishing a *Brucella* osteoarthritis animal model other than in mice is challenging.

Rats and guinea pigs are used commonly to assess vaccine efficacy and safety and both models have been investigated for brucellosis and *Brucella*-induced abortions. However, no studies have described utilization of these rodent models for studying brucellosis spondylitis (Bugybayeva et al., 2021; Hensel et al., 2020; Hensel et al., 2019; Lalsiamthara et al., 2019). Non-human primate models of *Brucella* infection have been reported in *Macaca arctoides* and *Macaca mulatta* (rhesus macaque) infected with *B. abortus*, *B. melitensis*, *B. suis*, or *B. canis* (Mense et al., 2004; Percy et al., 1972; Silva et al., 2011; Yingst et al., 2010). These animals are susceptible to infection with *Brucella* via oral, subcutaneous, or respiratory routes and exhibit persistent bacteremia for up to 8 weeks post-inoculation. Primate infection results in multiorgan disease characterized by focal granulomatous hepatitis, splenitis, and lymphadenitis which mirror the manifestations of human brucellosis. In certain instances, the reproductive system may be involved which leads to granulomatous orchitis, epididymitis, or acute endometritis (Mense et al., 2004). Aerosol infections also have been reported in nonhuman primates (Mense et al., 2004) with pathological alterations akin to human brucellosis which affirms the suitability of the nonhuman primate model for studying human brucellosis (Yingst et al., 2010). Unfortunately, there is a dearth of information concerning the use of non-human primates for developing models of brucellosis spondylitis, although models exhibiting symptoms similar to those of human brucellosis spondylitis have been established successfully in mice, rabbits, and dogs.

#### Rabbit model

A single study used rabbits to establish a brucellosis spondylitis model. This analysis replicated the radiological and histopathological characteristics of the human condition by inoculating live attenuated *Brucella* vaccine into the lumbar vertebrae of rabbits, which was followed 8 weeks later by changes in vertebral bodies and intervertebral discs, abscess formation within the paravertebral soft tissue, and a typical prominent inflammatory response, but without caseous necrosis, which closely resembled human *Brucellar* spondylodiscitis (Cai et al., 2019). The advantages of establishing a *Brucellar* spondylodiscitis model in rabbits include the fact that rabbits, as medium-sized animals, exhibit significant morphological and structural similarities with the human spine compared to small rodents such as mice and rats. These similarities allow for a more clear observation of the spine that facilitates exposure during surgery. Moreover, in comparison to large animals such as pigs, cows, and sheep, rabbits are more convenient for radiographic observations, as well as being easier and quicker to rear at a lower cost. The drawbacks of the rabbit model are that differences in innate immune responses exist between young and adult rabbits which result in varied outcomes in *Brucella* infection. In addition, the published study involved direct injection of *Brucella* into the lumbar vertebral bones of rabbits which established localized restrictive inflammation and lacked systemic bacterial dissemination. Furthermore, compared to species such as dogs, rats, and mice, the midsection of rabbit vertebral bodies is very narrow and primarily composed of cortical bone which renders intraosseous injection challenging. Additionally, the relatively narrow intervertebral spaces in rabbits do not facilitate high-dose *Brucella* injections (Cai et al., 2019).

#### Dog model

No studies have been described that constructed a brucellosis spondylitis model in dogs, although cases involving dogs naturally infected with *B. canis* that develop brucellosis discospondylitis have been presented (Forbes et al., 2019; Henderson et al., 1974; Kornegay and Barber, 1980; Long et al., 2022). For example, a retrospective analysis of 33 dogs, each of which had at least one positive *B. canis* test result or abnormal spinal imaging, revealed that 21/29 (72%) dogs presented with nonspecific pain, spinal pain, or lameness symptoms lasting up to 3 months. Only 4/28 (14%) exhibited fever symptoms. Multifocal lesions were evident on radiographs in 21/29 (72%) and by MRI in 12/18 (67%) of the animals. Smooth, round, central endplate lysis, defined as “hole punch” lesions, was identified radiographically in 25/29 (86%) of cases and 7/18 (39%) of the animals showed clear vertebral body inflammation or spondylitis without disc involvement by MRI (Long et al., 2022). Imaging of spondylitis and bilateral sacroiliitis also were described in a male hunting dog with a one-month history of lameness. Physical examination showed pain in the lumbosacral region and pelvis. Subsequent rapid slide agglutination and agar gel immunodiffusion tests confirmed *B. canis* infection in the animal (Forbes et al., 2019).

The preceding observations demonstrate that chronic pathology following *B. canis* infection in dogs may lead to canine *Brucella* osteoarthritis. However, this model has not yet been developed as dogs have a certain level of aggressiveness which may pose risks during experimental procedures compared to other animals. Furthermore, all reported cases of *Brucella* osteoarthritis in dogs have been caused by *B. canis* which is not the main species that causes human brucellosis thereby limiting the practical significance of the experiments for the human condition.

## Conclusion

*Brucella* osteoarthritis is the most common localized lesion in chronic human brucellosis and may result in the destruction of spinal structures. Spinal cord compression and partial loss of motor function may occur in severe cases which severely impacts the quality of life of patients. In-depth studies have been performed with individual clinical cases and imaging aspects of *Brucella* osteoarticular infections, as well as the development of relatively comprehensive diagnostic and treatment guidelines, have been undertaken. However, the mechanisms by which *Brucella* colonizes and causes chronic long-term infections in bone joints which leads to bone loss at these sites remain poorly documented and have been elucidated only in part. Furthermore, there is a lack of recognized and convincing animal models for *Brucella* spondylitis. This review has collated existing research data, and has summarized the interactions between bone matrix cells, including osteoblasts, osteoclasts, bone-resorbing cells, and local infiltrating inflammatory cells and inflammatory factors, with the aim of providing inspiration and guidance for subsequent studies on the pathophysiological mechanisms of *Brucella* osteoarthritis. The study of downstream mechanisms will promote the discovery of new treatment methods and may be used in conjunction with existing therapeutic approaches to prevent *Brucella* colonization of bone joints in the early stages of brucellosis and to inhibit bone loss, thereby reducing the incidence of osteoarticular *Brucella* infections.
